# Evaluating the effects of community-based organization engagement on HIV and AIDS-related risk behavior in Kenya

**DOI:** 10.1080/09540121.2013.778383

**Published:** 2013-06-09

**Authors:** Kara S. Riehman, Jakub Kakietek, Brigitte A. Manteuffel, Rosalía Rodriguez-Garcıa, Rene Bonnel, N'Della N'Jie, Lucas Godoy-Garraza, Alloys Orago, Patrick Murithi, Joseph Fruh

**Affiliations:** a American Cancer Society, Atlanta, GA, USA; b ICF Macro, Atlanta, GA, USA; c The World Bank, Washington, DC, USA; d National AIDS Control Council, Nairobi, Kenya

**Keywords:** HIV, community, community-based organization, prevention, Kenya

## Abstract

International donors have increasingly shifted AIDS funding directly to community-based organizations (CBOs) with the assumption that responding to the epidemic is best achieved at the community level. The World Bank, ICF Macro, and the National Council for Population and Development in Kenya, conducted a study to evaluate the community response in Kenya. The study used a quasi-experimental design comparing seven study communities and seven comparison communities in Nyanza Province and Western Province. We examined the impact of CBO activity on individual and community-level outcomes, including HIV knowledge, awareness and perceptions, sexual risk behavior, and social transformation (gender ideology and social capital). The study consisted of two components: a household survey conducted in all 14 communities, and qualitative data collected in a subset of communities. Individuals in communities with higher CBO engagement were significantly more likely to have reported consistent condom use. Higher CBO engagement was associated with some measures of social capital, including participation in local and national elections, and participation in electoral campaigns. CBOs provide added value in addressing the HIV and AIDS epidemic in very targeted and specific ways that are closely tied to the services they provide (e.g., prevention education); thus, increasing CBO engagement can be an effective measure in scaling up prevention efforts in those areas.

## Introduction

Civil society organizations (CSOs) and community-based organizations (CBOs)^1^ have been identified as essential to the global response to the AIDS pandemic (Schwartländer et al. 2011). At the local level, CBOs have been engaged in providing services to infected and affected people worldwide (UNAIDS, 2005). CBOs are also a key partner to governments in developing, implementing, and monitoring national responses to AIDS (UNAIDS, 2005). Over the last decade, international donors have increasingly shifted their AIDS funding in sub-Saharan Africa toward activities and programs implemented at the community level, particularly in low- and medium-income countries with a high HIV prevalence (UNAIDS, 2011).

The increased role of CBOs fighting AIDS was supported by the assumption that controlling and responding to the epidemic is best achieved through the active involvement of communities. Theoretical literature on CBOs has argued that they are well positioned to reach the previously missed elements of the society and tackle disease and poverty at their roots (Bratton, 1990; Clarke, 1998; Tendler, 1982). In addition to providing services such as care, support, and treatment, CBOs engage in local communities through other programs, such as conditional and unconditional cash transfers, income-generating activities, and microcredit schemes, especially the ones targeting marginalized groups with limited access to services and financial resources (Mohanty, 2006).

Empirical literature has shown that community-based interventions in Kenya have the potential to decrease the stigma associated with HIV/AIDS (Kaai et al., 2007; Waterman et al., 2007), improve access to treatment and treatment compliance (Amuyunzu-Nyamongo, Okeng'o, Wagura, & Mwenzwa, 2007; Ellis et al., 2006; Marston et al., 2007; McPeak, Doss, Barrett, & Kristjanson, 2009; Murphy, 2008; Selke et al., 2010), improve efforts to implement integrated multi-disease campaigns (e.g., HIV and TB, HIV and malaria; Granich, Muraguri, Doyen, Garg, & Williams, 2012; Kahn et al., 2011), or improve service delivery costs (Kahn et al., 2011). However, extant literature on the impact of community engagement on the response to HIV/AIDS in Kenya offers limited insights regarding the community-level impact of the activities carried out by community-based organizations. First, it tends to focus on assessing the effectiveness of a single intervention in a specific community setting, and does not address the effectiveness of a wide range of activities in a more general context. Second, it analyzes interventions carried out at the community level rather but not necessarily implemented by or through community-based organizations (World Bank, 2010b). Therefore, it offers only limited guidance regarding the key policy question facing the key global stakeholders in the fight against HIV/AIDS, namely, whether support and funding provided directly to local communities contributes to the increased availability and uptake of services, improved knowledge, and behavioral outcomes and, ultimately, adds value to the national and global responses to the epidemic.

To address this evidence gap, the World Bank commissioned a rigorous evaluation of the community response to HIV and AIDS in Kenya from January through September 2010. The evaluation addressed three primary research questions: Do community members in communities with stronger CBO engagement demonstrate (1) a significant difference in their access to and use of HIV and AIDS services; (2) a significant difference in knowledge, attitudes, perceptions, and behavior related to HIV and AIDS; and (3) a significant difference with respect to social transformation indicators compared to communities demonstrating weaker engagement?

### Context of the evaluation – HIV and AIDS epidemic in Kenya

Recent surveys estimate the prevalence of HIV in the adult population in Kenya to be between 6.3% and 7.4% (National AIDS/STD Control Program, Ministry Medical Services, 2007). The geographic distribution of HIV infections in Kenya is uneven, with the western provinces recording a higher prevalence of HIV than the eastern provinces (National ADIS/STI Control Program, Ministry Medical Services, 2007).

CBOs have been at the forefront of the fight against AIDS in Kenya. Their involvement was identified as a key component of the national response (KNASP, 2006–2009; Office of the President, National AIDS Control Council, 2009). A review of 37 studies of community-based interventions implemented in Kenya, however, found inconsistent evidence for an impact of CBO activity on HIV-related outcomes, given weak study designs and varying outcome metrics (World Bank, 2010a).

## Materials and methods

### Evaluation design and methodology

The evaluation used a quasi-experimental cluster design and multi-method data collection to compare the effects of CBO engagement on a set of identified outcomes between communities with higher and lower CBO engagement.^2^ In this study, community was defined as a collection of household units brought together by common interests, and/or made up of at least 5000 people (or 100 households for smaller villages living in the same geographical area. A total of 14 communities in two provinces (Nyanza and Western) and six districts Kisumu, Nyando, Kisii, Nyamira (Nyanza Province) and Butere-Mumias and Vihiga (Western Province) were included in the evaluation. A power analysis concluded that there would be 80% power to detect a medium effect size difference in 14 communities with a total of 192 households per community. Nyanza and Western provinces were selected for inclusion based on high HIV prevalence. The six districts have similar demographic characteristics, but varying levels of CBO activity. Selection of the communities occurred through several steps. First, the two provinces, Nyanza and Western, were selected for inclusion in the study based on high HIV prevalence. Within each province, the six districts that were selected were relatively close to one another and had relatively similar demographic characteristics, but were known to have varying levels of CBO activity. Within each district, a list of communities was delineated from the Kenya National Bureau of Statistics database. From this list, a random sample of 34 communities was selected. Communities in the random sample were assessed using specified criteria (See Table 1), assigned to study or comparison conditions (see below for assignment criteria), and matched based on similar demographic characteristics. To narrow the list to the final 14 communities, a matching process was undertaken in which a study community was paired with a comparison community. The equivalence of the communities when compared against the community selection protocol criteria was reviewed by representatives of the study team through intensive discussion, and was supplemented by verification of assumptions by consulting with relevant stakeholders familiar with the candidate communities. Particular criteria for equivalence were prioritized based on literature, seroprevalence survey findings and other relevant and available data in Kenya.

**Table 1.** List of criteria used to select communities that participated in the evaluation of the effects of the community response to *HIV*/AIDS in Kenya.

Level 1 criteria
1. The selected communities must each be identifiable as a community. It should be possible to geographically delineate each community and there should be some authoritative consensus on their classification as a community.
2. Both the study and the comparison community should be classified similarly with regard to their status as urban, suburban, periurban, or rural communities.
3. The study and comparison communities should be of similar size, as measured by number of households.
4. The two communities should be similar in terms of their language, and cultural and ethnic diversity or homogeneity. There should be no glaring systematic difference in this regard.
5. The communities should share a similar rate of employment.
6. The communities should share a similar level of income per household.
Level 2 criteria
1. There should be no significant differences between communities in the makeup and size of households.
2. The two communities should have a similar level of access to basic services, including water and sanitation, electricity supply, refuse removal and disposal, education, and any key social welfare services supplied by the state.
3. The nature and state of housing should be similar in the study and comparison communities.
4. Key health indicators, such as infant and maternal mortality rates, should be similar. The exception is seroprevalence rates, which do not have to be similar.
Level 3 criteria
The final matching procedure will examine the following criteria in addition to Levels 1 and 2.
1. Cultural values and practices: Paired communities will share the same dominant ethnic group, equalizing, for example, cultural practices such as female circumcision and cultural values tolerant of multiple sexual partners.
2. Prevalence of most-at-risk populations (MARPs): The proportionally higher infection rates of MARPs influence modes of transmission through bridging populations, for example, sex workers and their clients, the latter acting as a bridging vector for the disease into the rest of the population.
3. The comparative economic status of communities and the vector of economic growth: This would be related to access to basic services but more significantly the relative prevalence of MARPs, including migrant labor.
4. Proximity to major cities and commuting patterns: Not only does proximity to a major city correlate with higher levels of basic services and higher income levels, but there is the possibility of contagion as proximate communities take advantage of HIV and AIDS-related programs and services offered in the city, or city CBOs implement outreaches to neighboring communities.
5. Proximity to major transport routes: Both the general literature and case data indicate correlates with a higher prevalence of MARPs and the associated elevation of infection rates and risk of infection to the general population through bridging populations.

CBO activity level was measured as the number of active community-based organizations (CBOs) in the community and individual awareness of CBO activity in the community.

We used a database of CBOs maintained by the National AIDS Control Council (NACC) and information provided by local experts to capture the number of CBOs in each community. Given the potential that the NACC's CBO information was incomplete, and recognizing that the strength of the CBO activity may be determined by factors other than the number of CBOs, we used data collected from the household survey to provide additional information about the CBO reach, and to verify our initial study-comparison assignment. In the statistical analyses described below, the level of CBO engagement from the survey data was measured by the proportion of respondents who were aware of HIV/AIDS-related CBOs in their community. Communities with higher than the median proportion (46.9%) of respondents aware of services provided by CBOs were considered study communities, and communities with lower proportions of respondents aware of services provided by CBOs were considered comparison communities. Community assignment is not considered an absolute “high” versus “low,” but is rather a relative comparison of “higher” versus “lower” community engagement. A total of five communities were reclassified from the original assignment based on these post hoc criteria. Communities 9, 10, and 13 were reassigned from study to comparison, and communities 11 and 12 were reassigned from comparison to study. The final list of community assignment and their characteristics are presented in Table 2. All findings are presented for CBO engagement based on the post hoc assignment to the study and comparison conditions.

**Table 2.** Characteristics of the communities participating in the evaluation of the effects of the community response to *HIV/*AIDS in Kenya.

Province	District	Community	Community assignment	*HIV* prevalence*	*HIV* incidence (PMTCT)^a^	Rural/urban character	Main ethnic group	Population	Number of CBOs
Nyanza	Kisumu	1	Study	17.3	17.1	Urban	Mixed	10,000	10
		2	Study	17.3	17.1	Rural	Luo	2,000	10
		3	Study	17.3	17.1	Urban	Luo	4,000	10
	Nyando	4	Study	19	18.9	Rural	Luo	2,000	5
		5	Study	19	18.9	Rural	Luo	1,500	6
		6	Comparison	19	18.9	Urban	Luo	4,000	6
	Kisii	7	Comparison	5.9	7.2	Urban	Kisii	12,634	5
		8	Comparison	5.9	7.2	Rural	Kisii	6,027	2
	Nyamira	9	Comparison	5.2	4.0	Urban	Kisii	11,337	16
		10	Comparison	5.2	4.0	Rural	Kisii	8,676	14
Western	Butere-Mumias	11	Study	5.2	4.1	Urban	Luhya	9,545	5
		12	Study	5.2	4.1	Urban	Luhya	9,343	6
	Vihiga	13	Comparison	5.3	5.3	Urban	Luhya	6,307	6
		14	Comparison	5.3		Urban	Luhya	21,863	8

Note: ^a^Prevalence estimates available only at the district level.

### Household survey

A sample of 2715 households was randomly selected using official data for household populations from the list of National Sample Surveys and Evaluation Programme clusters, created and maintained by the Kenya National Bureau of Statistics (KNBS). In each household, up to three individual adult respondents were interviewed, resulting in a total of 4378 individuals. The household respondents were chosen based on selecting the head of household (if home at the time the interviewer went to the door), and the spouse of the household head (if existing and home). If the head of the household was not home, the interviewer talked with adults in the household and asked for nominations of up to three individuals. The head of household was asked to select other adult(s) to participate to reach up to three respondents. The Household Survey was administered to one individual per household, which included items assessing household-level constructs (e.g., living structure, household income, and government support). An individual questionnaire was administered to each individual respondent, which included individual-level constructs (e.g., HIV knowledge and awareness, sexual behavior). The survey was fielded from May to June 2010.

*Service awareness and utilization* was included in the individual survey and was measured using Yes-No questions whether the respondent was aware of (awareness questions) and used (utilization questions) services in the following categories: (1) prevention, (2) treatment, (3) care and support, (4) impact alleviation, and (5) community mobilization. The responses were coded as 1 if the respondent responded “Yes” and 0 otherwise.

*Knowledge* indicators reflected the UNGASS reporting system, and focused on the knowledge of HIV transmission modes, medications, and prevention. The individual survey included the following questions (with response options “Yes,” “No” or “Don't Know”): *People reduce HIV chances by having one uninfected partner; People reduce chances of getting HIV by using a condom; People CANNOT get the HIV virus from a mosquito or insect bite; People cannot get HIV by sharing utensils with a person who has AIDS; It is possible for a healthy-looking person to have HIV.* Correct responses were coded as 1 and incorrect (including “Don't Know”) as 0. In addition, respondents were asked the following Yes-No questions: *Are there any special drugs that a doctor or a nurse can give to a woman infected with the HIV virus to reduce the risk of transmission to the baby? Have you ever heard of VCT? Would you accept VCT if a volunteer came to the house? Do you know of drugs to control HIV?* The responses were coded as 1 if the respondent responded “Yes” and 0 otherwise. We analyzed each knowledge item separately because individual items measure different aspects of HIV- related knowledge (i.e., transmission, medication availability, and HIV testing). This mode of analysis provides a more nuanced assessment of the association between the community engagement and knowledge than using a single knowledge scale.

*Behavior* was measured using two indicators. – *consistent condom use with all partners in the past 12 months* and *ever having had an HIV test* (individual survey). Individuals were asked the number of sexual partners in the past 12 months, and for each partner, were asked, “Was a condom used every time you had sexual intercourse with that partner in the past 12 months?,” with response options Yes-No. Those who responded “Yes” to each partner were considered consistent condom users (coded “1”) and those who responded “No” to the question for any partner were not considered consistent condom users and coded “0”. Individuals were asked if they ever had an HIV test (response options Yes-No).

*Social transformation* was assessed using measures from the social science and public health literature focusing on social capital and gender norms. Social capital indicators were taken from the Adapted Social Capital Assessment Tool (A-SCAT) (Harpman, Grant, & Thomas, 2002). Social capital items were assessed at the household level. The following items were included: “In the last 12 months, how many members of your household (1) voted in a local election; (2) voted in a national election; (3) actively participated in an election campaign; (4) have taken part in a march or demonstration? How many institutions that protect children's rights are you aware of?” Respondents indicated total number of household members for each, and these items were coded as continuous variables.

*Cognitive social capital* was measured with a 20-item scale with items indicating perceived strength and cohesion of the community such as, “My neighbors would help me in an emergency; if someone in the community lost their job, there are community members who would help them with money or other resources.” Response categories included “Strongly Disagree, Disagree, Agree, and Strongly Agree.” A mean and summative scale was created and analyzed as separate outcomes. A scale of attitudes toward one's own children was measured using six items (Yes-No response): “Do you help your children to understand that they have their rights; Do you help your children to understand that their rights are legitimate and nobody can violate them; Do you help your children to react against the violation of their rights; Do you discuss with your children when others’ rights are violated; Do you encourage your children to react against violation of others’ rights; and Do you allow your children to participate in decision-making in the family?” “Yes” responses were coded “1,” and “No” responses were coded “0.” An average score across all items was calculated.

Indicators of *gender norms* were based on surveys developed by the Kenya Diffusion and Ideational Change Project and the Malawi Diffusion and Ideational Change Project (Watkins, Behrman, Kohler, & Zulu, 2003). For this analysis, gender ideology was measured with two items: “Do you think it's right for women to use modern family planning if they want to stop childbearing or to have a rest between children;” and “Do you use modern family planning?” with response options Yes-No (only females responded to this question; thus, the analysis for this outcome included only females).

Multilevel hierarchical models were estimated to account for potential autocorrelation from clustered data (e.g., individuals within households, households within communities), using Mplus (Muthen & Muthen, 2010). Multilevel logistic regression models were estimated for dichotomous variables and multilevel logistic regression models were estimated for continuous outcomes.

Bivariate differences in demographic factors between study and comparison communities resulting from post hoc reassignment of communities were found for HIV prevalence, rural versus urban characters, employment, age, and marital status (Table 3). These differences were controlled in the multivariate analysis.

**Table 3.** Summary statistics of study and comparison communities.

Communities	Study (High level of CBO engagement) *n* = 7 (%)	Comparison (Low level of CBO engagement) *n* = 7 (%)
HIV prevalence	14.3	7.4
Gender distribution		
Male	40.1	39.1
Female	59.9	60.9
Ever attended school	96.8	95.5
Engaged in paid work	69.9	45.3
Marital status		
Married	70.5	77.3
Divorced	4.1	3.4
Widowed	7.4	6.1
Never married	18.1	13.2

In addition to those factors, we also controlled for education as this variable is typically associated with HIV knowledge and behavior. For binomial outcomes, logistic regression was performed, and results are reported as odds ratios with confidence intervals. For continuous outcomes, linear regression was performed, and results are reported as coefficients with standard errors and p-values.

### In-depth interviews with the CBOs and KIs

In-depth semi-structured interviews with 28 CBOs and 58 key informants (KIs) were conducted in a sub-sample of eight communities. The interviews provided more detailed information on CBO services and activities, and data on social transformation and contextual factors that facilitate and constrain the effectiveness of HIV activities and programs. Participating CBOs were identified based on the NACC database of CBOs and supplemented by information provided by the NACC staff and local community experts. KIs included (1) the director of the Constituency AIDS Control Committee (a local arm of the NACC) in each community, (2) a local government leader, (3) the head nurse from the largest local clinic, (4) a senior clergy person from the largest religious congregation, and (5) the principal of the largest school.

The interviews with CBO staff focused on the activities of CBOs, their targets/audiences, geographic reach, and staff and volunteers involved. Questions included the following: ‘When was the organization established? What did the response to the HIV/AIDS epidemic look like before your organization was established? What activities did your organization focus on initially? How has the scope of activities changed over time? What goals have you achieved in your community? What are some of the factors that have helped you achieving your goals? How do you think your work has affected the way women/orphans/people with HIV are treated? What is your experience with the local government response to HIV/AIDS? How does your organization interact with government? How does your organization interact with other local organizations?’ The interviews with KIs provided information about social capital and transformation in the community, and the perceived role of CBOs in affecting those factors. Questions included “Over the past five years what changes have you observed in how people trust each other/voting behavior/participation in voluntary organizations/collective action/the ability of women to make economic decisions/girls’ access to education/violence against women/how people with HIV are treated? What factors do you think caused any changes?”

Trained interviewers conducted the interviews in the local language, and took detailed notes, and in some cases, recorded, and transcribed the interviews. Notes and transcription were translated into English. Interviews were reviewed and coded by a team of two individuals. Initial codes were developed to match the interview topics, and additional codes were added during the review and coding process as needed.

## Results

### Household survey – HIV/AIDS-related outcomes

Significant associations found between the key independent variables – higher (coded “1”) or lower (coded “0”) CBO engagement – and measures of HIV knowledge, attitudes, perceptions, behavior, service utilization, and social transformation are in bold in the tables.

### Knowledge and behavior

Table 4 shows results of the models assessing HIV knowledge and behavior. The level of CBO engagement was positively associated with several indicators. Respondents in the study group had more than nine times higher odds than respondents in the comparison group of knowing that having one uninfected partner reduces the chances of HIV transmission (OR = 9.26, 95% CI = 5.29–16.22), and almost 15 times higher odds of knowing that using a condom reduces the chances of becoming infected with HIV (OR = 14.67, 95% CI = 10.58–20.35). Respondents in the study group had about three times higher odds of knowing that the chances of vertical transmission of HIV can be reduced by prevention of mother-to-child transmission (PMTCT) (OR = 3.84, 95% CI = 2.70–5.48). Respondents in the study group were also more likely to know that one cannot get HIV from sharing utensils with a person with HIV (OR = 1.76, 95% CI = 1.29–2.44), more likely to have ever heard of VCT (OR = 1.89, 95% CI = 1.00–3.56), and more likely to know of drugs to control HIV (OR = 3.54, 95% CI = 1.74–7.19).

**Table 4.** Results of multilevel regression analysis HIV knowledge and risk behavior.

	People reduce HIV chances having 1 uninfected sex partner^a^		People reduce chances of getting HIV by using a condom^a^		Know of drugs to reduce MTC transmission^a^		People CANNOT get the HIV virus from a mosquito or insect bite^a^		People cannot get HIV by sharing utensils with a person who has AIDS^a^		It is possible for a healthy looking person to have HIV^a^		Have heard of VCT^a^		Would accept VCT if a counselor came to the house^a^		Know of drugs to control HIV^a^		Condom use^a^		Ever tested for HIV^a^	
Variables	(OR)	95% CI	(OR)	95% CI	(OR)	95% CI	(OR)	95% CI	(OR)	95% CI	(OR)	95% CI	(OR)	95% CI	(OR)	95% CI	(OR)	95% CI	(OR)	95% CI	(OR)	95% CI
Age	1.02	(1.01–1.03)	1.00	(0.99–1.00)	0.99	(0.98–1.00)	0.99	(0.99	− 1.00)	1.00	(0.99–1.00)	0.99	(0.98–1.00)	0.96	(0.95–0.98)	0.99	(0.98–0.99)	0.99	(0.98–0.99)	0.96	(0.94–0.98)	0.95	(0.95–0.96)
Female	0.85	(0.71–1.02)	0.65	(0.57–0.73)	1.92	(1.61–2.28)	0.68	(0.59	− 0.78)	1.04	(0.94–1.15)	0.73	(0.64–0.83)	0.66	(0.42–1.03)	1.19	(0.98–1.44)	0.96	(0.80–1.16)	0.74	(0.64–0.86)	1.98	(1.73–2.26)
Number of school	0.68	(0.47–0.97)	0.56	(0.45–0.70)	0.66	(0.51–0.86)	0.50	(0.42	− 0.60)	0.52	(0.41–0.65)	0.39	(0.30–0.50)	0.24	(0.19–0.31)	0.72	(0.51–1.02)	0.81	(0.53–1.22)	0.34	(0.09–1.24)	0.45	(0.35–0.58)
Secondary school	1.01	(0.84–1.21)	1.36	(1.17–1.58)	1.80	(1.5–2.17)	2.27	(1.94	− 2.66)	1.53	(1.32–1.76)	1.66	(1.39–1.99)	3.00	(2.17–4.15)	1.10	(0.95–1.28)	1.61	(1.20–2.16)	2.07	(1.51–2.82)	1.42	(1.26–1.60)
College	2.35	(1.57–3.50)	2.32	(1.72–3.14)	1.38	(1.01–1.90)	7.37	(5.32	− 10.23)	3.44	(2.50–4.73)	5.78	(3.62–9.23)	4.20	(2.19–8.05)	1.27	(0.94–1.72)	1.78	(1.12–2.84)	1.72	(1.10–2.67)	2.57	(2.01–3.28)
Divorced/separated	1.19	(0.84–1.69)	1.39	(1.07–1.79)	1.10	(0.88–1.37)	1.34	(1.10	− 1.62)	0.88	(0.65–1.19)	1.67	(1.06–2.62)	0.91	(0.46–1.79)	1.16	(0.79–1.71)	1.27	(0.83–1.94)	72.31	(23.43–223.18)	0.68	(0.48–0.97)
Widowed	0.80	(0.58–1.10)	0.98	(0.81–1.20)	1.10	(0.88–1.37)	1.08	(0.90	− 1.30)	1.10	(0.99–1.22)	1.52	(1.21–1.90)	0.96	(0.69–1.33)	0.88	(0.69–1.12)	3.22	(1.97–5.24)	72.89	(21.71–244.69)	0.93	(0.75–1.14)
Never married and never lived together	1.37	(0.99–1.88)	1.34	(1.08–1.68)	0.59	(0.48–0.72)	0.85	(0.71	− 1.02)	0.84	(0.73–0.96)	0.84	(0.70–1.02)	1.03	(0.61–1.76)	0.78	(0.66–0.91)	0.75	(0.54–1.04)	300.37	(115.01–784.46)	0.29	(0.24–0.36)
Engaged in any paid work	1.19	(0.95–1.50)	0.81	(0.68–0.97)	1.13	(0.96–1.34)	0.99	(0.86	− 1.12)	1.14	(0.94–1.39)	0.89	(0.78–1.02)	1.21	(0.80–1.81)	1.27	(1.08–1.5)	1.54	(1.19–1.98)	1.17	(0.91–1.49)	1.15	(1.01–1.30)
Household wealth index	0.97	(0.8–1.19)	1.05	(0.88–1.26)	1.34	(1.11–1.60)	1.19	(1.06	− 1.33)	1.34	(1.15–1.56)	1.13	(0.98–1.30)	0.85	(0.63–1.16)	0.81	(0.74–0.89)	1.06	(0.80–1.41)	0.80	(0.68–0.93)	1.08	(0.97–1.19)
Rural	1.03	(0.57–1.86)	1.32	(0.98–1.78)	1.32	(0.86–2.02)	0.80	(0.59	− 1.09)	0.94	(0.70–1.26)	0.89	(0.63–1.26)	0.49	(0.33–0.72)	1.07	(0.71–1.60)	0.19	(0.05–0.69)	0.35	(0.23–0.53)	0.70	(0.57–0.86)
HIV prevalence	0.95	(0.91–0.99)	0.95	(0.92–0.99)	0.98	(0.96–0.99)	0.98	(0.96	− 0.99)	0.96	(0.93–0.99)	0.98	(0.96–1.01)	1.15	(1.09–1.22)	1.03	(1.00–1.06)	1.16	(1.08–1.24)	1.16	(1.13–1.18)	1.09	(1.07–1.11)
Community assignment	**9.26**	**(5.29–16.22)**	**14.67**	**(10.58–20.35)**	**3.85**	**(2.70–5.48)**	1.09	(0.92	− 1.28)	**1.78**	**(1.29–2.44)**	1.27	(0.96–1.67)	**1.89**	**(1.00–3.56)**	1.02	(0.70–1.48)	**3.54**	**(1.74–7.19)**	**4.09**	**(3.05–5.48)**	**1.53**	**(1.23–1.92)**

Notes: ^a^Binomial outcome variable model.

Condom use = used condom consistently (with all sex partners) at last sex (12 months).

In addition, respondents in the study group had four times higher odds of reporting consistent condom use with all sex partners in the last 12 months (OR = 4.09, 95% CI = 3.05–5.49), and were more likely to have ever had an HIV test (OR = 1.54, 95% CI = 1.23–1.92).

### Service awareness and utilization

We found no statistically significant association between the strength of CBO engagement and service utilization.

### Social transformation – social capital and gender ideology

Tables 5 and 6 present models assessing social capital and gender ideology. Respondents in the study group significantly had more household members who were aware of institutions that promote and protect children's rights (ß = 1.25, SE = 0.39). CBO engagement was also significantly associated with three indicators of institutional social capital capturing civic engagement and political participation. In the study group, households had an average of almost two more people who voted in national and in local elections than households in the comparison group, and 1.3 more persons who participated in electoral campaigns (ß = 1.71, SE = 0.86; ß = 1.83, SE = 0.56; ß = 1.28, SE = 0.33, respectively).

**Table 5.** Results of multilevel regression analysis: social capital.

	Know of institutions that protect children's rights^a^		Voted in local election^a^		Voted in general election^a^		Participated in electoral campaign^a^		Scale of attitudes towards own children^a^		Respect opinion of children^a^		Participated in community activities^a^		Taken part in a march or demonstration^a^		Cognitive social capital mean score^a^		Cognitive social capital summative score^a^		Participation in voluntary associations^a^	
Variables	Coefficient (SE)	*P* value	Coefficient (SE)	*P* value	Coefficient (SE)	*P* value	Coefficient (SE)	*P* value	Coefficient (SE)	*P* value	Coefficient (SE)	*P* value	Coefficient (SE)	*P* value	Coefficient (SE)	*P* value	Coefficient (SE)	*P* value	Coefficient (SE)	*P* value	Coefficient (SE)	*P* value
Age	0.00 (0.00)	0.41	0.00 (0.00)	0.33	0.00 (0.00)	0.83	−0.01 (0.01)	0.35	0.01 (0.01)	0.41	0.02 (0.01)	0.02	0.00 (0.00)	0.04	0.00 (0.00)	0.75	0.00 (0.00)	0.28	−0.01 (0.01)	0.28	0.00 (0.01)	0.70
Number of school	−0.42(0.19)	0.03	0.45 (0.24)	0.06	0.28 (0.61)	0.64	0.82 (0.44)	0.06	−0.43 (0.29)	0.13	−0.20 (0.18)	0.28	0.07(0.19)	0.72	0.68 (0.56)	0.23	0.04 (0.05)	0.43	1.25(0.89)	0.16	−0.61 (0.32)	0.06
Secondary school	0.69(0.11)	0.00	0.20(0.13)	0.15	0.23 (0.12)	0.07	0.06 (0.19)	0.77	0.54 (0.09)	0.00	0.44 (0.10)	0.00	0.20 (0.09)	0.02	0.34 (0.21)	0.10	−0.04(0.02)	0.02	−0.96(0.37)	0.01	0.40 (0.08)	0.00
College	1.80(0.24)	0.00	0.74 (0.32)	0.02	0.77 (0.37)	0.04	0.87 (0.21)	0.00	0.97(0.18)	0.00	0.87 (0.17)	0.00	0.54 (0.09)	0.00	−0.31 (0.49)	0.52	−0.09(0.03)	0.00	−1.75(0.50)	0.00	0.60(0.16)	0.00
Divorced/separated	−0.16(0.16)	0.31	−0.90 (0.29)	0.00	−0.84(0.37)	0.02	−0.71 (0.26)	0.01	−0.56 (0.21)	0.01	−0.25 (0.29)	0.39	−0.46(0.23)	0.05	−0.76 (0.57)	0.18	0.11 (0.04)	0.00	2.38 (0.72)	0.00	−0.52(0.19)	0.01
Widowed	0.06(0.14)	0.68	−0.66(0.14)	0.00	−0.74(0.18)	0.00	−0.45(0.20)	0.02	0.28 (0.23)	0.24	0.52(0.14)	0.00	−0.15(0.07)	0.05	−0.52(0.31)	0.09	−0.02(0.04)	0.63	−0.46(0.68)	0.50	0.20(0.12)	0.09
Never married and never lived together	−0.59(0.12)	0.00	−0.11 (0.16)	0.52	−0.11 (0.14)	0.44	−0.47(0.24)	0.05	−1.37(0.25)	0.00	−0.94 (0.23)	0.00	−0.26(0.09)	0.01	−0.08 (0.28)	0.79	0.09 (0.03)	0.00	1.74(0.57)	0.00	−0.61 (0.19)	0.00
Engaged in any paid work	0.33 (0.09)	0.00	−0.24(0.18)	0.20	−0.74(0.19)	0.00	−0.01 (0.20)	0.95	0.25 (0.09)	0.01	0.20 (0.18)	0.26	0.08(0.10)	0.43	0.29 (0.21)	0.17	−0.01 (0.02)	0.81	−0.04 (0.42)	0.92	0.26(0.13)	0.04
Household wealth index	0.14(0.10)	0.13	−0.27(0.13)	0.04	−0.10(0.27)	0.70	−0.07(0.17)	0.67	0.19(0.10)	0.05	0.00 (0.11)	0.97	−0.24(0.06)	0.00	0.03 (0.11)	0.77	−0.02(0.02)	0.40	−0.35 (0.40)	0.39	−0.06(0.10)	0.57
Rural	−0.15(0.32)	0.64	0.90 (0.52)	0.08	1.90(0.85)	0.03	0.06 (0.57)	0.92	4.87 (0.39)	0.00	−0.19 (0.74)	0.80	0.01 (0.19)	0.96	0.03 (0.40)	0.93	0.18(0.03)	0.00	69.62 (12.21)	0.00	−0.06(0.39)	0.88
HIV prevalence	0.00 (0.03)	0.95	−0.02 (0.04)	0.57	−0.07(0.09)	0.40	0.06 (0.04)	0.13	0.01 (0.07)	0.93	0.04 (0.07)	0.59	0.08 (0.01)	0.00	0.05 (0.02)	0.03	−0.00(0.01)	0.82	−0.03 (0.17)	0.88	0.05 (0.03)	0.10
Community assignment	**1.25** (0.39)	**0.00**	1.83 (0.56)	**0.00**	1.71 (0.86)	**0.05**	**1.28** (0.33)	**0.00**	−0.60 (0.81)	0.46	−0.35 (0.75)	0.64	0.04(0.15)	0.79	0.38 (0.27)	0.16	−0.21 (0.11)	0.07	−4.04 (2.30)	0.08	0.38 (0.32)	0.23

Note: ^a^Ordinal outcome variable model.

**Table 6.** Results of multilevel regression analysis: gender ideology.

	Gender ideology 1^a^		Gender ideology 4^a^	
Variables	(OR)	95% CI	(OR)	95% CI
Age	0.99	(0.98–1.00)	0.97	(0.96–0.98)
Female	3.00	(2.65–3.40)	0.86	(0.61–1.22)
Number of school	0.41	(0.31–0.55)	1.02	(0.89–1.17)
Secondary school	1.71	(1.48–1.97)	1.24	(1.00–1.54)
College	1.92	(1.56–2.38)	0.46	(0.38–0.56)
Divorced/separated	1.02	(0.70–1.49)	0.22	(0.15–0.31)
Widowed	0.65	(0.52–0.81)	0.40	(0.28–0.57)
Never married and never lived together	0.50	(0.42–0.61)	1.13	(0.98–1.31)
Engaged in any paid work	1.28	(1.10–1.49)	1.17	(1.02–1.34)
Household wealth index	1.24	(1.14–1.35)	1.38	(1.05–1.81)
Rural	1.16	(0.94–1.41)	0.97	(0.95–1.00)
HIV prevalence	0.95	(0.93–0.96)	1.25	(1.04–1.50)
Community assignment	1.10	(0.90–1.33)	0.97	(0.96–0.98)

Notes: ^a^Binomial outcome variable model.

Gender ideology 1 = it's right for women to use modern family planning.

Gender ideology 4 = uses modern family planning method (women only).

Strength of CBO engagement was not associated with cognitive social capital or any indicators of gender norms.

### In-depth interviews: CBO activity and social transformation

#### CBO activity

The average length of time a CBO was present in the community ranged between 5.5 and 8.8 years. The average number of volunteers in the past year ranged from 4 to 26. The average number of CBO clients reported in a community varied widely, and ranged from 67 to 853.

CBOs were engaged in similar activities across study and comparison communities. In all eight communities, CBOs engaged in prevention activities, support for PLWHA, activities targeting OVC, and information and education activities aimed at increasing the awareness and knowledge of HIV and AIDS. In seven communities, CBOs also engaged in care; in five communities, in advocacy efforts; and in three communities, in impact mitigation. CBOs engaged in provision of medical services in only one community. In six communities, CBO leaders mentioned behavior change communication as part of their organization's mission, and in three communities, CBOs distributed condoms. The number of the clients reached as a percentage of the overall population was the most pronounced difference between the study and the comparison communities (2.7% and 0.5%, respectively).

#### Social transformation

The interviews provided historical depth to the quantitative survey and insight into how social capital, gender relations, and stigma and discrimination have changed over time. Furthermore, the interviews with KIs were designed to help determine the extent to which any observed differences in outcomes between study and comparison communities could be attributed to the activities of local CBOs.

### Voting

Voting, an indicator of social capital in terms of civic engagement, was reported by KIs to have increased in all communities – they noted that turnouts in national and local elections have increased over the past five years. Most attributed the increase to rising education levels and higher awareness of political and voting rights among community members. They did not, however, identify what caused the shifts in education and awareness, and none explicitly mentioned CBO activity as the cause of those changes. While KIs did not identify CBOs as a driving force behind increased voting behavior, survey data showed that this indicator was significantly higher in higher engagement communities.

### Participation in voluntary organizations

In all communities, most of the KIs noted increases in participation in voluntary organizations within the past five years. Self-help groups and groups engaging in income-generating activities, such as setting up fishing ponds or communal poultry-raising, were mentioned as the most common forms of organizations emerging in the research communities. In one community, KIs noted that people formed neighborhood associations assisting families in organizing and financing funerals. The fact that individuals from both study and comparison communities noted increased participation in organizations supports the lack of differences found in this measure of social capital by the household survey.

In three communities, increases in participation were attributed to normative changes, although no agents driving the normative change were identified. In one community, CBOs were explicitly mentioned as the source of change. KIs noted that the CBOs in the community have “sensitized people on the importance of coming together.” In the same community, two other KIs attributed the recent changes to increase in government assistance given to voluntary associations. In one community, increases in the number of voluntary organizations and in the number of community members belonging to such organizations were attributed to the local leadership's support of such initiatives.

### Gender norms

In three communities, KIs reported an increase in economic opportunities available to women. In two communities, the KIs said that women were gaining more influence over how to spend the household money. In one community, some KIs attributed this to activities of self-help groups engaging in income-generating activities.

In addition, respondents noted structural factors such as women being more aware of their rights, general increases in the level of education among women in Kenya, and migration (in households where the man worked outside of the community, women were thought to have more influence over how the household money is being spent).

Across all communities, KIs noted increases in the number of girls enrolled in elementary schools. They attributed the main drivers of the increases in female enrollment in education and also perceived declines in violence against women to shifts in national policies, rather than CBO activity. KIs credited the CBOs with helping to offset some of the expenses related to education, such as paying for school uniforms or providing financial support for orphans to prevent them from dropping out. With respect to women's economic agency and their influence over household finances, KIs in two communities mentioned income-generating activities carried out by CBOs as increasing women's access to money, but not necessarily in increasing their influence on how to spend it.

When asked explicitly about the impact of the CBOs on gender relations in their communities, KIs credited the CBOs with helping to offset some of the expenses related toeducation, suchaspaying for school uniforms or providing financial support for orphans to prevent them from dropping out. With respect to violence against women, KIs in all communities mentioned that CBOs educated women about their rights. In one community, one KI noted that a CBO also provided legal representation for victims of violence. The interviews, however, suggest that those activities hadalimited impactand wereofsecondary importance compared to the changes in national policies.

The qualitative data regarding gender roles support the findings from the survey, in that there were no statistically significant differences between the study and comparison communities in measures of gender norms in the survey. This is consistent with perceptions of the KIs that changes gender norms have occurred across the country as a result of national policies, rather than CBO activity.

## Discussion

The objective of this evaluation to assess, using a scientifically rigorous methodology, whether CBOs providing HIV and AIDS prevention and treatment activities add value to the government response to the epidemic and have a measurable impact on individual and community-level outcomes. While the study did not assess changes in HIV incidence or prevalence, it was able to identify how higher CBO engagement and activity affects some aspects of knowledge and behavior that partially drive the epidemic.

People in communities where a high proportion of individuals were aware of CBO activity showed greater knowledge of some HIV transmission and prevention methods, were more likely to use condoms consistently, and ever have been tested for HIV, demonstrated greater likelihood to engage in community mobilization activities, including voting and participating in electoral campaigns. While CBOs provided similar services across the study and comparison communities, what differentiated communities was the number of community members aware of, and, possibly, reached by these activities. Thus, in communities where CBOs are engaging in prevention efforts, and higher numbers of community members are aware of their work, the levels of knowledge and awareness may be higher, and people may be more likely to engage in lower risk sexual behaviors.

The qualitative data provide insight into the larger national effort in Kenya to address the social drivers of the HIV/AIDS epidemic. For example, national policies that have increased access to education seem to have made a significant impact on increasing the rights of women in all communities in the evaluation, regardless of existing CBO activity. It is notable that some key informants viewed CBOs as increasing awareness of policies and laws through outreach and awareness campaigns. Thus, the potential for CBOs to augment national efforts can be viewed as a means of disseminating information from the national to the local level.

The study findings are subject to several limitations. First, the lack of data on CBO activities made a priori classification of the community response difficult. To address this limitation, measures of CBO engagement embedded in the household survey served to verify accuracy of a priori group assignments. The community-matching process and controlling, through multivariate analysis, for potentially confounding factors that differed across communities, reduced the likelihood of factors other than CBO engagement driving the associations detected in the analyses.

The evaluation was conducted at one point in time, limiting the ability to make causal conclusions about the impact of CBO activity on the outcomes of interest. For example, the fact that individuals in some communities are more aware of CBO services may be the driving force behind, rather than the outcome of, a high level of CBO activity in that community. Similarly, greater awareness and political activism may make it easier for CBOs to establish themselves and be successful in a specific community. While the cross-sectional design does not make it possible to reach causal conclusions, the evaluation was designed with these limitations in mind. Additional evidence from qualitative interviews helped support the conclusions obtained from the quantitative analysis. Our analysis included testing several outcome variables, which may increase the risk of type 1 error. However, it also increases the level of detail and nuance of the overall analysis.

We did not conduct sensitivity analysis to assess how the results presented above change if we change the cut-off we used to assign communities to study and comparison communities. The same cut-off for assignment was used in all three components of the study: the household survey, the interviews with the KIs, and the interviews with the CBO staff. This allowed us to show that the CBOs in both groups engaged in similar types of activities (in the study communities, more people were exposed to the activities in which the CBO engaged) and, from the KI interviews, that the contextual factors across both groups of the communities were roughly the same and the only difference between the study and comparison group was the intensity of exposure to CBO activity. Changing the cut-off point we used in the analysis of the household survey data would require reanalysis of the data from the interviews with CBO staff, to assess whether there were no systematic differences in the types of activities in which the CBOs engaged in the reassigned groups, and reanalysis of the interviews with the KIs, to assess whether there were no systematic differences in contextual factors across the newly assigned study and comparison groups. We consider the multi-method design to be the key strength of our study that allows for triangulation of information across the quantitative and qualitative components. However, it makes sensitivity analysis of a single study component exceptionally challenging. Triangulation from multiple data sources enabled us to distinguish instances in which better HIV/AIDS-related outcomes could be attributed to CBO activities from outcomes that were most likely affected by other factors, including policy changes at the national level. This evaluation suggests that CBOs provide added value in addressing the HIV/AIDS epidemic in targeted and specific ways that are closely tied to the services they provide. Increasing CBO engagement can be an effective measure in scaling up prevention efforts, especially those aimed at improving knowledge and awareness of AIDS in Kenya.
